# Supplementary material for: Multistate Markov Model to Predict the Prognosis of High-Risk Human Papillomavirus-Related Cervical Lesions
Source: Cancers (Basel). 2020 Jan 22;12(2):270. doi: 10.3390/cancers12020270 (PMC7072567; doi:10.3390/cancers12020270)
Supplement: Supplementary file 1 [file cancers-12-00270-s001.pdf]

Article

Multistate Markov Model to Predict the Prognosis of High-Risk Human Papillomavirus-Related Cervical Lesions

Ayumi Taguchi, Konan Hara, Jun Tomio, Kei Kawana, Tomoki Tanaka, Satoshi Baba, Akira Kawata, Satoko Eguchi, Tetsushi Tsuruga, Mayuyo Mori, Katsuyuki Adachi, Takeshi Nagamatsu, Katsutoshi Oda, Toshiharu Yasugi, Yutaka Osuga and Tomoyuki Fujii

Table S1. Predicted 2-year transition probabilities from CIN1 to CIN2 or more severe lesions and their 95% confidence intervals for the six HPV categories derived from the Cox regression analysis.

HPV category	2-year transition probability
HPV16	0.502 (0.232–0.676)
HPV18	0.086 (0.000–0.235)
HPV52	0.527 (0.336–0.662)
HPV58	0.498 (0.251–0.663)
Other hrHPVs	0.253 (0.144–0.349)
No hrHPVs	0.133 (0.065–0.197)

HPV, human papillomavirus; hrHPV, high-risk human papillomavirus.Cox proportional hazard model was used to predict 2-year transition probability from CIN1 to CIN2 or more severe lesions for each HPV category. To this end, we restricted the patients to those who were diagnosed as CIN1 at the time of entry and followed them until they were diagnosed as CIN2 or more severe lesions or at the end of their observation period. The parameters for hazards for the HPV categories were independently estimated. In cases of co-infection, patients could contribute to the parameter estimation of several HPV categories. Based on the estimated parameters, we simulated the probabilities of transitioning from CIN1 to CIN2 or more severe lesions after 2 years.

Table S2. Sample size and summary statistics for the combination of patients in the six HPV categories and diagnoses at the time of entry in the dataset for the alternative model.

Diagnosis at the time of entry		HPV 16	HPV 18	HPV 52	HPV 58	Other hrHPVs	No hrHPVs	All
Normal	N	13	11	17	13	30	122	195
	Age at the time of entry (years), mean (SD)	42.4 (13.8)	39.5 (15.0)	36.7 (10.0)	41.3 (16.8)	42.5 (16.9)	41.2 (10.5)	41.3 (12.1)
	Number of visits, mean (SD)	6.7 (6.1)	8.7 (4.6)	8.8 (7.2)	8.0 (5.6)	7.0 (4.8)	6.6 (3.9)	7.0 (4.6)
	Follow-up interval (years), mean (SD)	0.47 (0.33)	0.47 (0.28)	0.48 (0.34)	0.46 (0.39)	0.47 (0.27)	0.52 (0.39)	0.49 (0.35)
	Follow-up period (years), mean (SD)	2.7 (2.6)	3.4 (2.0)	4.1 (3.3)	3.3 (2.5)	2.9 (2.2)	3.1 (2.1)	3.2 (2.3)
CIN1	N	23	11	38	24	79	111	259
	Age at the time of entry (years), mean (SD)	34.6 (8.2)	33.0 (10.0)	36.6 (8.6)	36.1 (7.7)	34.5 (7.2)	39.0 (10.3)	36.9 (9.2)
	Number of visits, mean (SD)	8.6 (5.4)	7.6 (4.7)	11.2 (6.1)	10.5 (6.3)	9.3 (4.9)	9.4 (5.2)	9.6 (5.2)
	Follow-up interval (years), mean (SD)	0.40 (0.39)	0.48 (0.48)	0.38 (0.17)	0.45 (0.43)	0.39 (0.21)	0.42 (0.31)	0.41 (0.29)
	Follow-up period (years), mean (SD)	3.5 (2.5)	3.2 (2.2)	4.2 (2.4)	3.9 (2.6)	3.5 (2.1)	4.0 (2.3)	3.8 (2.3)
CIN2/CIN3	N	147	24	94	83	103	78	459
	Age at the time of entry (years), mean (SD)	36.6 (7.8)	39.9 (6.8)	40.1 (8.0)	38.7 (7.7)	38.2 (8.1)	37.2 (8.7)	38.4 (8.1)
	Number of visits, mean (SD)	7.3 (6.2)	9.3 (7.1)	8.2 (6.3)	9.8 (5.9)	8.9 (5.9)	9.3 (5.9)	8.5 (6.1)
	Follow-up interval (years), mean (SD)	0.31 (0.22)	0.32 (0.14)	0.34 (0.20)	0.35 (0.22)	0.36 (0.31)	0.35 (0.31)	0.34 (0.25)
	Follow-up period (years), mean (SD)	2.1 (2.2)	3.0 (2.9)	2.7 (2.5)	3.5 (2.5)	3.1 (2.3)	3.4 (2.4)	2.8 (2.4)

CIN, cervical intraepithelial neoplasia; HPV, human papillomavirus; hrHPV, high-risk human papillomavirus; SD, standard deviation. Cytological and histological results were combined to classify the results into the following diagnoses: normal, CIN1, and CIN2/CIN3. HPVs 16, 18, 31, 33, 35, 39, 45, 51, 52, 56, 58, 59, and 68 were classified as hrHPVs. Of these, HPV 16, 18, 52, and 58 were categorized separately. hrHPVs other than HPVs 16, 18, 52, and 58 were classified as "other hrHPVs." Patients who were not infected with any hrHPVs were referred to as "no hrHPVs" patients. In the cases of observed co-infection with different HPV genotypes, it was possible to include the same patient in the summary statistics of multiple HPV categories.

Table S3. Summary of the transitions from each diagnosis of cervical epithelial lesions for the six HPV categories in the dataset for the alternative model.

		**Diagnosis at t^th^ visit**			
**Diagnosis at (t-1)th visit**	**HPV category**	**Normal**	CIN1	CIN2/CIN3	Treatment
Normal	HPV 16	289 (83.2)	18 (5.1)	37 (10.6)	3 (0.8)
	HPV 18	123 (82.0)	16 (10.6)	8 (5.3)	3 (2.0)
	HPV 52	322 (75.4)	62 (14.5)	43 (10.0)	0 (0.0)
	HPV 58	284 (79.3)	38 (10.6)	34 (9.4)	2 (0.5)
	Other hrHPVs	687 (85.7)	79 (9.8)	30 (3.7)	5 (0.6)
	No hrHPVs	1381 (89.7)	116 (7.5)	37 (2.4)	4 (0.2)
CIN1	HPV 16	34 (25.9)	40 (30.5)	55 (41.9)	2 (1.5)
	HPV 18	23 (40.3)	21 (36.8)	13 (22.8)	0 (0.0)
	HPV 52	85 (31.8)	109 (40.8)	70 (26.2)	3 (1.1)
	HPV 58	55 (30.8)	73 (41.0)	48 (26.9)	2 (1.1)
	Other hrHPVs	141 (40.6)	144 (41.4)	55 (15.8)	7 (2.0)
	No hrHPVs	213 (53.9)	137 (34.6)	44 (11.1)	1 (0.2)
CIN2/CIN3	HPV 16	54 (7.6)	58 (8.1)	505 (71.2)	92 (12.9)
	HPV 18	14 (9.2)	12 (7.9)	113 (74.8)	12 (7.9)
	HPV 52	60 (11.7)	67 (13.1)	332 (65.0)	51 (10.0)
	HPV 58	52 (9.9)	52 (9.9)	375 (71.9)	42 (8.0)
	Other hrHPVs	68 (13.3)	68 (13.3)	335 (65.8)	38 (7.4)
	No hrHPVs	77 (22.3)	43 (12.4)	205 (59.4)	20 (5.7)

CIN, cervical intraepithelial neoplasia; HPV, human papillomavirus; hrHPV, high-risk human papillomavirus. Values are the number (percentage) of transitions observed from prior diagnosis to current diagnosis for each HPV category in the dataset for the alternative model. Cytological and histological results were combined to classify the results into the following diagnoses: normal, CIN1, and CIN2/CIN3. HPVs 16, 18, 31, 33, 35, 39, 45, 51, 52, 56, 58, 59, and 68 were classified as hrHPVs. Of these, HPV 16, 18, 52, and 58 were categorized separately. hrHPVs other than HPVs 16, 18, 52, and 58 were classified as "other hrHPVs." Patients who were not infected with any hrHPVs were referred to as "no hrHPVs" patients. In the cases of observed coinfection with different HPV genotypes, it was possible to include the same patient in multiple HPV categories.

Table S4. Predicted 2-year transition probabilities from states to states and their 95% confidence intervals for the six HPV categories in the alternative model.

Current state	HPV category	State after 2 years			
		Normal	CIN1	CIN2/CIN3	Treatment
Normal	HPV 16	0.543 (0.461–0.619)	0.081 (0.064–0.102)	0.230 (0.187–0.275)	0.144 (0.110–0.186)
	HPV 18	0.608 (0.493–0.706)	0.137 (0.098–0.184)	0.184 (0.121–0.252)	0.068 (0.037–0.118)
	HPV 52	0.501 (0.441–0.556)	0.183 (0.157–0.209)	0.218 (0.184–0.257)	0.096 (0.071–0.128)
	HPV 58	0.520 (0.459–0.584)	0.151 (0.127–0.176)	0.246 (0.201–0.291)	0.081 (0.057–0.108)
	Other hrHPVs	0.703 (0.657–0.744)	0.142 (0.122–0.163)	0.111 (0.090–0.136)	0.042 (0.030–0.057)
	No hrHPVs	0.824 (0.798–0.848)	0.104 (0.090–0.119)	0.054 (0.043–0.069)	0.016 (0.010–0.024)
CIN1	HPV 16	0.367 (0.303–0.433)	0.069 (0.055–0.088)	0.247 (0.205–0.291)	0.315 (0.260–0.376)
	HPV 18	0.528 (0.421–0.624)	0.128 (0.091–0.173)	0.212 (0.145–0.289)	0.131 (0.074–0.213)
	HPV 52	0.439 (0.382–0.489)	0.172 (0.146–0.198)	0.227 (0.193–0.264)	0.161 (0.124–0.207)
	HPV 58	0.439 (0.380–0.501)	0.141 (0.118–0.166)	0.272 (0.224–0.318)	0.147 (0.109–0.192)
	Other hrHPVs	0.624 (0.576–0.666)	0.140 (0.120–0.160)	0.135 (0.111–0.164)	0.099 (0.073–0.133)
	No hrHPVs	0.787 (0.756–0.813)	0.105 (0.092–0.121)	0.065 (0.051–0.082)	0.042 (0.027–0.061)
CIN2/CIN3	HPV 16	0.254 (0.203–0.306)	0.060 (0.047–0.076)	0.242 (0.197–0.288)	0.442 (0.380–0.509)
	HPV 18	0.335 (0.240–0.441)	0.100 (0.067–0.140)	0.259 (0.169–0.363)	0.304 (0.195–0.449)
	HPV 52	0.342 (0.293–0.388)	0.149 (0.125–0.173)	0.225 (0.188–0.265)	0.283 (0.224–0.353)
	HPV 58	0.322 (0.272–0.373)	0.122 (0.100–0.145)	0.291 (0.238–0.342)	0.263 (0.203–0.336)
	Other hrHPVs	0.465 (0.411–0.515)	0.128 (0.109–0.148)	0.162 (0.132–0.200)	0.243 (0.190–0.309)
	No hrHPVs	0.668 (0.611–0.716)	0.104 (0.090–0.119)	0.085 (0.064–0.113)	0.140 (0.096–0.200)

CIN, cervical intraepithelial neoplasia; HPV, human papillomavirus; hrHPV, high-risk human papillomavirus. Values are the predicted probabilities (95% confidence intervals) of transitions from the current state to the state after 2 years for each HPV category. Cytological and histological results were combined to classify the results into the following diagnoses: normal, CIN1, CIN2, and CIN3. HPVs 16, 18, 31, 33, 35, 39, 45, 51, 52, 56, 58, 59, and 68 were classified as hrHPVs. Of these, HPV 16, 18, 52, and 58 were categorized separately. hrHPVs other than HPVs 16, 18, 52, and 58 were classified as "other hrHPVs." Patients who were not infected with any hrHPVs were referred to as "no hrHPVs" patients. In the cases of observed coinfection with different HPV genotypes, it was possible to include the same patient in multiple HPV categories. We used the continuous-time multistate Markov model to estimate the prognosis of each patient. In the alternative model, we defined four states: normal (state 1), CIN1 (state 2), CIN2/CIN3 (state 3), and treatment (state 4). Arrows in Figure S2 specify possible transitions between the states defined in our model; all transitions between adjacent states, except the backward transition from treatment to CIN2/CIN3, were allowed. CIN3/cancer was the absorbing state. Treatment was the absorbing state. We truncated observations after the treatment intervention.

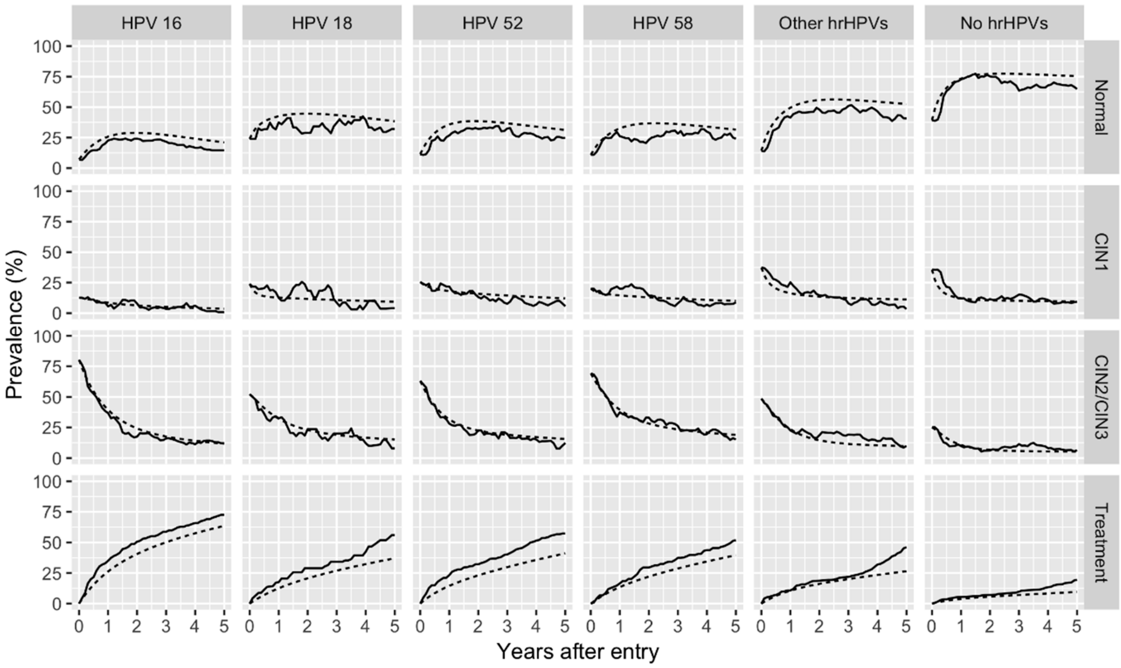

Figure S1. Observed and simulated prevalence transition of each state for the human papillomavirus (HPV) categories in the alternative model. The figure demonstrates the observed (solid line) and simulated (dotted line) prevalence transition of each state for the six HPV categories in the alternative model. Cytological and histological results were combined to classify the results into the states of the model. HPVs 16, 18, 31, 33, 35, 39, 45, 51, 52, 56, 58, 59, and 68 were classified as high-risk HPVs (hrHPVs). Of these, HPV 16, 18, 52, and 58 were separately categorized. hrHPVs other than HPVs 16, 18, 52, and 58 were classified as "other hrHPVs." Patients who were not infected with any hrHPVs were referred to as "no hrHPVs" patients. In cases of observed coinfection with different HPV genotypes, it was possible to include the same patient in multiple HPV categories.

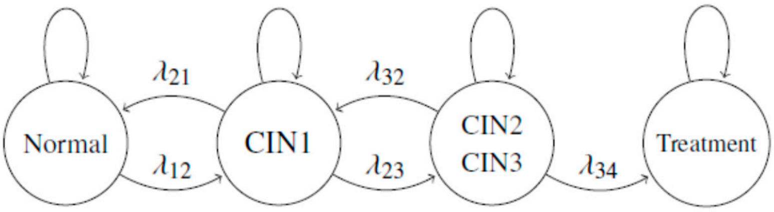

Figure S2. Markov model for the disease progression and regression of cervical epithelial lesions of the alternative model. The figure displays the schema of the Markov model for the alternative model. We defined four states: normal (state 1), cervical intraepithelial neoplasia 1 (CIN1, state 2), CIN2/CIN3 (state 3), and treatment (state 4). The arrows in the figure specify possible transitions between these states; all transitions between adjacent states, except the backward transition from treatment to CIN2/CIN3, were allowed. Treatment was the absorbing state. Each transition parameter λ indicates the transition intensity; i.e., λij is interpreted as an "instantaneous risk" of transition from state i to j.

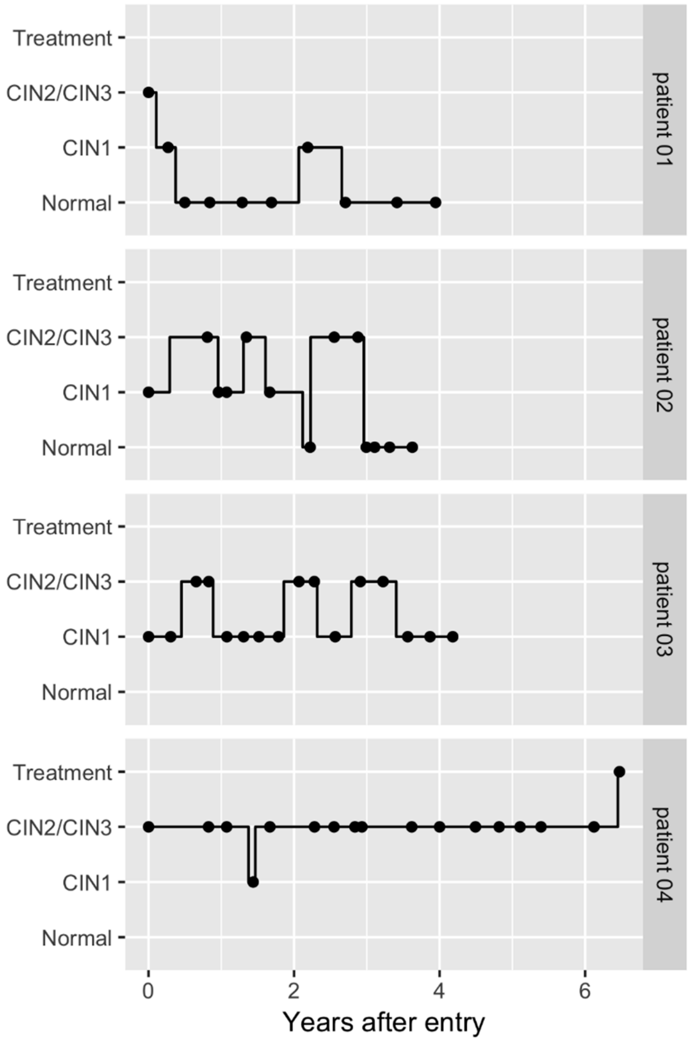

Figure S3. Possible transition paths of the alternative model for the selected patients. The figure shows possible transition paths for four selected patients (patient 01–patient 04). Cytological and histological results were combined to classify the results into the states of the model. We truncated observations after the diagnosis of treatment intervention in this alternative model. The filled circles indicate actual observations or visits. The solid line is a possible transition path during the follow-up period. The possible transition paths were randomly selected on the basis of the observed states.
